# Benign Multicystic Peritoneal Mesothelioma: A Rare Condition in an Uncommon Gender

**DOI:** 10.1155/2017/9752908

**Published:** 2017-05-18

**Authors:** Muhammad S. Khurram, Hamadullah Shaikh, Uqba Khan, Jacob Edens, Warda Ibrar, Ameer Hamza, Awais Zaka, Roohi Bano, Tarik Hadid

**Affiliations:** ^1^St. John Hospital and Medical Center, Detroit, MI 48236, USA; ^2^Champion Orthopedics, Hamilton, NJ, USA; ^3^Henry Ford Macomb Hospital, Clinton Township, MI, USA

## Abstract

Benign Multicystic Peritoneal Mesothelioma (BMPM) is a rare condition that arises from the abdominal peritoneum. Fewer than 200 cases have been reported worldwide. BMPM usually affects premenopausal women and is extremely rare in men. Many factors are suspected to contribute to its development, such as previous surgery, endometriosis, and familial Mediterranean fever. The main management is surgical resection; however, it is estimated that the recurrence rate is up to 50%. Malignant transformation is rare. We report a case series of three male patients who were diagnosed with BMPM and were treated with cytoreductive surgery and hyperthermic intraperitoneal chemotherapy (HIPEC).

## 1. Introduction

Benign Multicystic Peritoneal Mesothelioma is a rare benign tumor that presents with recurrent peritoneal mesothelial cysts that arise from the epithelial and mesenchymal elements of mesothelial tissue. It has an extremely low potential of transformation to malignant mesothelioma. The incidence of this disease is 0.15/100,000 annually, which makes it challenging to diagnose and treat [1]. To date, there are 140 reported cases in literature. Due to its rarity, the definitive prognosis of BMPM is not yet established. However, from the limited cases reported, the prognosis appears to be very good and only one death has been reported: a 14-year-old patient who died 12 years after diagnosis due to refusal of surgical intervention [2–5].

BMPM was first described in 1928 by Plaut, who incidentally observed loose pelvic cysts during an operation for uterine leiomyoma. The mesothelial origin was later confirmed by electron microscopy [6]. A few reports proposed a possible genetic or familial predisposition for BMPM. Specifically, one case report, published by Curgunlu et al. [7], describes a man with familial Mediterranean fever who developed BMPM. Although familial Mediterranean fever is linked to development of malignant mesothelioma, the patient was found to have BMPM without malignant mesothelioma.

BMPM is more common in women of reproductive age, with mean age of 36 years. In female patients, there appears to be an association between BMPM and endometriosis, pelvic inflammatory disease, and previous abdominal surgeries. In male patients, previous abdominal surgeries were linked to development of BMPM. The mean age in male patients in our case series is 67 years. As the disease is rarely reported in males, the variation in mean age between male and female patients needs to be determined [1, 3, 6].

## 2. Clinical Summary

The first patient was a 68-year-old Caucasian male who underwent routine ventral and inguinal hernia repair; however during the procedure he was incidentally found out to have BMPM. Patient history was significant for hypertension, long duration of smoking, and exposure to asbestos while working in the automotive industry. He underwent a CT scan of the chest, abdomen, and pelvis, which revealed a large cystic mass filling the right hemiabdomen and the pelvis; however no abnormality was seen in chest. The patient underwent cytoreductive surgery and HIPEC without any major postoperative complications. The second case is a 72-year-old African American male, who presented with hematuria and underwent a CT scan of the chest, abdomen, and pelvis that revealed a 10 × 8 × 4.7 cm right lower quadrant cystic mass along with ascites, with no findings in chest. Patient medical history was significant for early stage prostate cancer treated with prostatectomy. The patient was a former smoker but did not have any exposure to asbestos. Family history was significant for colon and breast cancer. Patient underwent cytoreductive surgery and HIPEC. Our third case was a 61-year-old Caucasian male that presented with abdominal pain. The patient underwent a CT scan of the chest, abdomen, and pelvis, which revealed a 10 cm cystic mass in the right lower abdominal quadrant with no abnormality noticed in chest. Patient medical history was significant for coronary artery disease, stroke, hypertension, and remote history of mucocele of the appendix, status after appendectomy. The patient denied smoking history and there was no significant asbestos exposure. Family history was significant for lung cancer and leukemia. The patient underwent cytoreductive surgery and HIPEC, which revealed involvement of BMPM with small bowel, cecum, colon, and peritoneum. The patient had uneventful postoperative period.

## 3. Pathologic Findings

**Figure 1:**
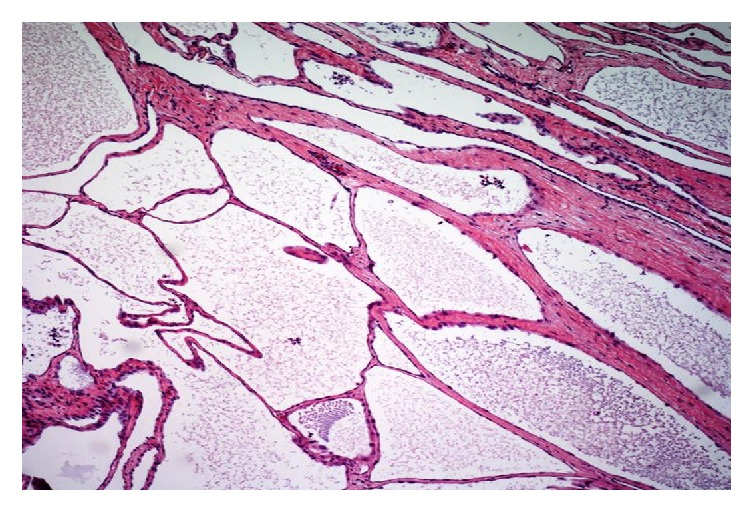
H&E-Low power magnification showing soft tissue with numerous variably sized cystic spaces.

**Figure 2:**
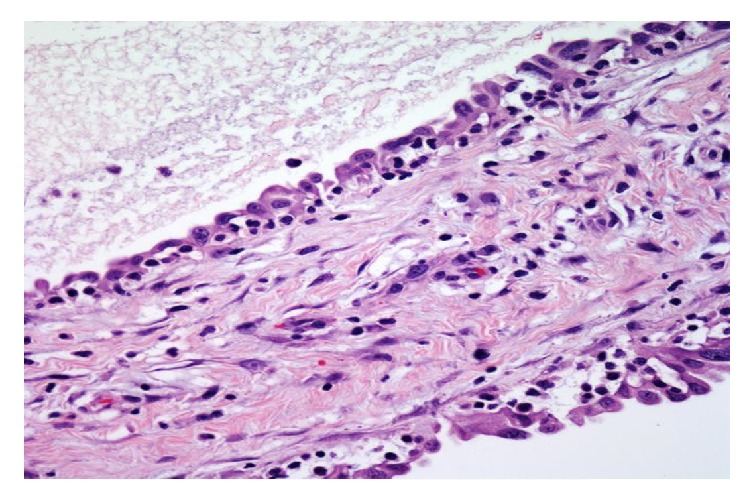
Cyst lining composed of single layer of mesothelial cells (H&E 40x).

**Figure 3:**
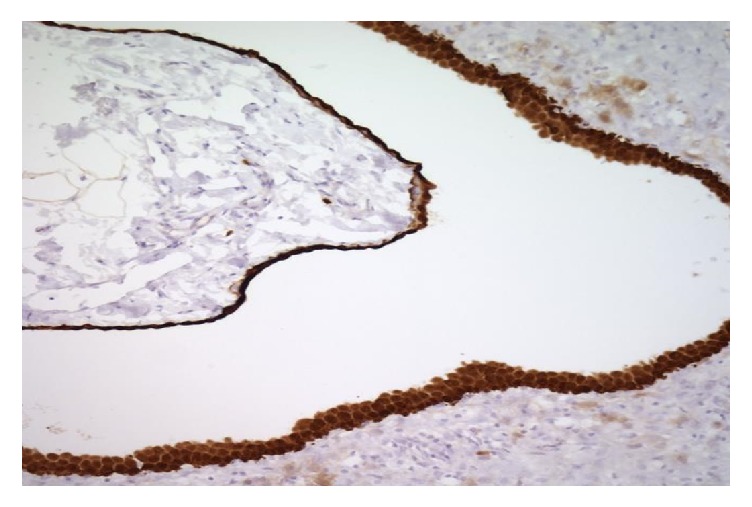
Calretinin positive cells lining the cyst wall.

**Figure 4:**
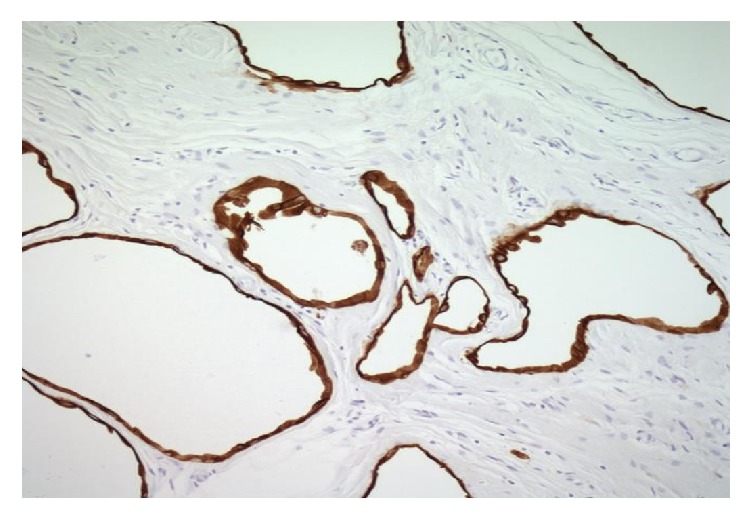
Cyst lined by cytokeratin AE1/AE3 positive cells.

The surgical specimen from all three patients was sampled thoroughly with approximately 2 sections per centimeter. They demonstrated multiple cysts (Figure 1) with thin walls, lined with single layer of flattened-to-cuboidal mesothelial cells (Figure 2) and filled with serous fluid. The cells were diffusely positive for calretinin (Figure 3), cytokeratin AE1/AE3 (Figure 4), and EMA. In immunohistochemical analysis, positive expression of calretinin by the mesothelial cells is always seen [7, 8].

## 4. Discussion

The etiology and pathogenesis of BMPM remain unclear. However, three hypotheses have been proposed. Some authors believe that BMPM derives from chronic inflammatory processes involving the peritoneum, which results in a reactive hyperplastic and dysplastic transformation of mesothelial cells. Others suggest a more primitive neoplastic origin of the peritoneal serosa without strict association with coexistent chronic inflammatory insult. Another theory is a hormonal hypothesis, in which the development and progression of BMPM are tightly linked to its sensitivity to sex hormones. This theory is supported by the evidence of higher incidence in women during their reproductive age and the responsiveness of BMPM to certain endocrine agents such as tamoxifen and gonadotropin-releasing hormone analogs [9]. Two of our cases had a family history of colon, breast, and lung cancer and leukemia, respectively, suggesting more reporting of this entity. Despite the lack of universal consensus, most authors adopt the first theory in which chronic peritoneal inflammation occurs, which results in proliferation and migration of peripheral mesothelial cells often associated with metaplasia of the underlying connective tissues [9–11]. The symptoms of BMPM are usually vague and occur mostly when the tumor is quite large causing mass effect on other organs. These may include chronic or intermittent abdominal and/or pelvic pain, abdominal fullness, abdominal distention, intestinal obstruction, unintentional weight gain, and changes in bowel habits [8, 12, 13]. Physical examination may reveal abdominal tenderness, abdominal distention, and/or a palpable abdominal or pelvic mass(es). Unusual symptoms can occur and may simulate serious conditions such as acute appendicitis. One case report published by Safi Khuri describes an unusual case of a 19-year-old man who presented with abdominal pain in right lower quadrant (RLQ) for 2 days, accompanied by diarrhea. His physical examination was unremarkable except for RLQ tenderness. Abdominal ultrasound and computed tomography (CT) revealed a multicystic mass. Diagnostic laparoscopy confirmed the diagnosis of BMPM [14]. One of our patients presented with similar symptoms but with prior history of appendectomy ruling out the diagnosis of acute appendicitis. CT scan showed a large cystic lesion in the RLQ and diagnostic laparoscopy confirmed the diagnosis of BMPM.

The majority of patients with BMPM are asymptomatic until the disease affects other organs. In the two cases described here, the first patient's lesion was incidentally found during the patient's hernia surgery. The second patient presented with clinical symptoms and signs suggestive of acute appendicitis but since he had prior appendectomy, other diagnoses were sought. Imaging studies are needed to characterize the disease with CT scan being the imaging modality of choice, which usually reveals multiple abdominal cysts. Therefore, laparoscopy remains the best diagnostic tool as it is minimally invasive and enables us to obtain specimens to establish the definitive diagnosis, which relies primarily on histological examination [8, 14].

There are malignant and benign disorders that can simulate BMPM. These primarily include Lymphangioma or Malignant Peritoneal Mesothelioma. Lymphangioma can be distinguished when cysts consist predominantly of chylous fluid, lymphoid aggregates, smooth muscle, and CD2-40 positive expression on immunohistochemical analysis. Malignant Peritoneal Mesothelioma occasionally presents with a history of asbestos exposure (BMPM is almost never associated with asbestos exposure), abdominal pain, distention, and weight loss. An abdominal CT may show significant ascites and diffuse peritoneal thickening [8, 12]. Although the potential for transformation to malignancy is very low, there are two reported cases of malignant transformation. De Stephano et al. described a 6-month-old infant who was diagnosed with a cystic lesion involving the liver parenchyma. The infant expired 11 months following partial resection of this lesion. He was found to have focal residual disease and diffuse parietal and visceral peritoneal studding on autopsy. González-Moreno et al. also reported a 36-year-old woman with a ten-year history of BMPM. She underwent bilateral ovarian cystectomies and partial omentectomy with peritoneal washing, followed by five additional laparoscopies for recurrent and persistent ascites over 10 years. Despite conservative management, she developed recurrent umbilical hernia, tense ascites, and umbilical fistula [15]. During the sixth laparoscopic exploration, she was found to have malignant mesothelioma arising from a benign cystic lesion. The inguinal, internal, and external iliac, para-aortic, and inferior deep epigastric and celiac lymph nodes were found to be involved. It was also noted that benign and malignant lesions coexisted at the same time. Our first and second case had no recurrence of cystic disease on surveillance CT after two years of diagnosis. Our third case was recently diagnosed in December 2016. He recovered well during his postsurgical period and was allowed to return to work without any restrictions.

Multiple treatment strategies have been attempted to treat BMPM. Yet, complete cytoreduction followed by instillation of hyperthermic intraperitoneal chemotherapy (HIPEC) remains the treatment of choice. This modality is thought to lower the recurrence rate and allow removal of microscopic residual tumor. However, the recurrence rate of BMPM remains high at approximately 50% in women and 33% in men even after complete cytoreduction. For surveillance, some authors suggest obtaining a CT scan every 3 months for the first year after resection and then annually for the next 5 years. This may result in early detection of relapse, which allows less invasive surgical resection and may decrease the risk of malignant transformation [12, 13, 16]. However, the impact of this strategy on overall survival is yet to be established.

Various chemotherapeutic agents have been used intraoperatively. Different cancer centers have established their protocols for HIPEC. Agents used are chosen based on the type of malignancy and the experience of the surgical oncologist. The most common combination of drugs used in this disease is cisplatin and doxorubicin [17]. All of our patients received HIPEC following complete surgical excision and they remained asymptomatic without any recurrence of BMPM.
